# Integrated Circuit Board Object Detection and Image Augmentation Fusion Model Based on YOLO

**DOI:** 10.3389/fnbot.2021.762702

**Published:** 2021-11-11

**Authors:** Szu-Yin Lin, Hao-Yu Li

**Affiliations:** ^1^Department of Computer Science and Information Engineering, National Ilan University, Yilan City, Taiwan; ^2^Department of Information Management, Chung Yuan Christian University, Taoyuan City, Taiwan

**Keywords:** smart manufacturing, Internet of Things, deep learning, YOLO, object recognition

## Abstract

Industry 4.0 has been a hot topic in recent years. The process of integrating Cyber-Physical Systems (CPS), Artificial Intelligence (AI), and Internet of Things (IoT) technology, will become the trend in future construction of smart factories. In the past, smart factories were developed around the concept of the Flexible Manufacturing System (FMS). Most parts of the quality management process still needed to be implemented by Automated Optical Inspection (AOI) methods which required human resources and time to perform second stage testing. Screening standards also resulted in the elimination of about 30% of the products. In this study, we sort and analyze several Region-based Convolutional Neural Network (R-CNN) and YOLO models that are currently more advanced and widely used, analyze the methods and development problems of the various models, and propose a suitable real-time image recognition model and architecture suitable for Integrated Circuit Board (ICB) in manufacturing process. The goal of the first stage of this study is to collect and use different types of ICBs as model training data sets, and establish a preliminary image recognition model that can classify and predict different types of ICBs based on different feature points. The second stage explores image augmentation fusion and optimization methods. The data augmentation method used in this study can reach an average accuracy of 96.53%. In the final stage, there is discussion of the applicability of the model to detect and recognize the ICB directionality in <1 s with a 98% accuracy rate to meet the real-time requirements of smart manufacturing. Accurate and instant object image recognition in the smart manufacturing process can save manpower required for testing, improve equipment effectiveness, and increase both the production capacity and the yield rate of the production line. The proposed model improves the overall manufacturing process.

## Introduction

Smart manufacturing is based on smart factories involving artificial intelligence (AI), the Internet of Things (IoT), big data, and other technical tools. Smart manufacturing is the general term referring to an advanced manufacturing process and a system capable of perceiving information intuitively, making decisions automatically, and executing manufacturing processes automatically (Wang et al., 2018). In addition, it reports the current status of each device through the process of mechanical automation. Statistics and summarizing data can help us understand the device's condition or estimate its usable period. Moreover, smart manufacturing combines machines and deep learning technology to improve product quality and reduce costs. Consequently, the machinery has attained better production efficiency and adaptive maintenance time within the effective period. Providing better or more flexible services to customers is part of smart manufacturing's pursuit of true intelligence. Smart manufacturing is the focus of recent Industry 4.0 topics related to research and development or industry promotion. However, there are several issues in the implementation of smart manufacturing. Before the topic of smart manufacturing was formally proposed, the core concept in the background of automated manufacturing was the flexible manufacturing system (FMS) (Kimemia and Gershwin, 1983; Bihi et al., 2018). FMS hoped to establish a flexible and automated manufacturing engineering system in response to all predictable or unpredictable changes in the industry. However, this goal can only be achieved with the assistance of other technologies or systems (Yadav and Jayswal, 2018). In a process related to quality management inspection, although automated optical inspection (AOI) is applied, the screening standards are too high, and approximately 30% of the products are eliminated (Mukhopadhyay et al., 2019; Kovrigin and Vasiliev, 2020; Diering and Kacprzak, 2021). Moreover, this method requires a massive workforce and time to perform inspection in the second stage. In addition, only through the operator's correct implementation of various standard inspection procedures can it guarantee accurate manufacturing quality management. Therefore, a large number of professional employees undergo long-term training, increasing the labor cost. Smart manufacturing should include automated perception at its core and find a way to attain automated intelligence ultimately. In the process, various technologies and methods, such as intelligent image recognition and intelligent data analysis, can help achieve automatic identification and prediction. Auxiliary decision-making can also be used to perform automated execution in the environment, though it will be challenging.

As AI image recognition becomes more and more mature nowadays, the combination of deep learning with classic computer vision has become a trend. Today, most mainstream technology for image recognition applications uses convolutional neural networks (CNNs). Since the re-emergence of deep learning in 2012, scholars and experts have proposed several new methods to solve the problems encountered by neural networks in the past. The shortcomings of CNNs in the past have also been reduced (Khan et al., 2020). In recent years, the characteristics of graphics processing units have also been fully utilized to accelerate the calculation of deep learning algorithms; therefore, the algorithm's efficiency has dramatically improved. The most crucial technological turning point in image recognition is the development of the region-based convolutional neural networks (R-CNN) algorithm. This technology first solved the problem of the insufficient dataset, and later, the related models introduced also performed well in terms of performance and recognition accuracy (Bharati and Pramanik, 2020). Based on it, the Faster R-CNN algorithm was developed, which allows the calculation speed of the algorithm to reach a different level of sophistication. As a result, image recognition technology is getting closer and closer to the goals of achieving both high speed and high precision (Gavrilescu et al., 2018; Maity et al., 2021).

Nowadays, several cases of the combination of computer vision with deep learning of the IoT have been implemented, and many positive feedbacks have been obtained in academic research and real-life applications (Wang et al., 2020; Xu et al., 2020; Lian et al., 2021). Accurate image recognition technology helps classify product types, confirm product integrity in an actual field, and helps establish a smart manufacturing field. The method proposed in this study is based on the R-CNN-related model of the deep learning method. The integrated circuit board (ICB) image is selected as the dataset to complete the image recognition model. The first stage aims to acquire different types of ICB images for model training. Thus, we first constructed the initial phase of image recognition so that the model can understand the characteristics of different types of ICBs and their details. In the second stage, a camera is used for real-time identification of the smart manufacturing field by collecting real-time images and returning the data to the server for data analysis, thereby solving the FMS's quality management inspection and monitoring. This study has three main objectives: (1) to establish an image recognition model that is suitable for use in the smart manufacturing field; (2) to explore the image augmentation fusion and optimization method of the model so that the model can learn more image features to improve the accuracy of image recognition; and (3) to solve the problem of over screening in automatic optical inspection and introduce the model into practical applications to test the directionality of ICB images.

## Literature Reviews

### R-CNN and SPP-Net

There are three main problems to be solved by region-based convolutional neural networks (R-CNN), which involve (1) accuracy of object recognition; (2) whether more feature values can be obtained; and (3) solving the problem of insufficient dataset. Compared with previous CNNs, R-CNN proposes a method for selecting region proposals of selective search (Girshick et al., 2014) to increase its dataset and find critical features. Previously, when solving dataset problems, the data augmentation method mentioned in “ImageNet Classification with Deep Convolutional Neural Networks” was first considered (Krizhevsky et al., 2012). Notably, the R-CNN region proposal's concept also aims at this problem (Girshick et al., 2014). In R-CNN, the input of selective search (Girshick et al., 2014) is an image, and the output is the possible position of the object. The principle is to initialize a similar empty set in advance, calculate the similarity of all adjacent intervals, store it in the empty set, find the region with the highest degree of similarity, and return it to the final total set. The region in the total set is the object's bounding box, and the similarity is judged based on color, texture, size, and shape, and iteratively combining similar regions to form objects. R-CNN obtains many region proposal images through the selective search method, but still needs to use the same image size as the input of the entire neural network because the fully connective layer in CNN must maintain the exact dimensions in operation, and the operation parameters also need to consider the upper-layer relationship. However, spatial pyramid pooling network (SPP-Net) addressed this issue: by adding a layer of SPP before building the fully connective layer. The function and principle of SPP are that the data process is performed before regular data input to comply with the fully connective layer problems mentioned above. SPP replaces the last pooling layer before the fully connective layer, and to adapt to the feature maps of different resolutions, the layer is defined as a scalable pooling layer so that a fixed ratio can be used through SPP. This way of converting and maintaining the input of the fully connective layer is a breakthrough in this part (He et al., 2014).

### Fast R-CNN and Faster R-CNN

The core problem with R-CNN is that it generates a large number of region proposal images through selective search. When pre-processing data, we still have to refer to the data augmentation method by AlexNet (Krizhevsky et al., 2012) to make modifications, which may lead to the loss of features of the original region proposal. In addition, if each region proposal image is put into training, the vast computational waste caused by repeated feature extraction will make the model inefficient. With SPP-Net, it is still time-consuming to train all the images, so Fast R-CNN was created. Fast R-CNN is a unified version of R-CNN and SPP-Net. Fast R-CNN proposed RollPooling (region of interest pooling), which uses the idea of SPP-Net to do the conversion work into the fully connective layer based on the input image. First, the original image is convolved to generate a feature map corresponding to RollPooling. Then the image trained in the region proposals is directly given the convolution value of the region proposal image through RollPooling to do MaxPooling. The most significant advantage of RollPooling is the increase of massive processing speed. Besides, regardless of the size of the given feature map, the dimensions of the output data can be kept uniform (Girshick, 2015). The key problem Fast R-CNN (Girshick, 2015) wants to solve is calculating the image of the region proposals. Hence, an improved Faster R-CNN was developed to solve the issue of repetitive region proposals directly. It does not abandon the selective search (Girshick et al., 2014) method but finds region proposals with features more efficiently. Therefore, the concept of RPN (region proposal network) is proposed in the Faster R-CNN architecture. The core concept of RPN is not to find the region proposals from the original image but to find the region proposals through the convolved feature map of the original image as input. The RPN extracts region proposals through a sliding window, and each sliding window generates nine different size of windows (anchor box). After removing the corresponding nine window features, the extra part is discarded, and the anchor box with an overlap area value >0.7 as the foreground is calculated. The overlapping area is set to the background, the most suitable region proposals feature map is found, and the concept of RollPooling is combined to train the model. This method is very similar to Fast R-CNN in terms of results and has dramatically improved the speed. It is also one of the most commonly used models in R-CNN (Ren et al., 2016).

### YOLO

After introducing Faster-RCNN (Ren et al., 2016), You Only Look Once (YOLO) (Redmon et al., 2016) and ordinary R-CNN were introduced in the same year with different architectures. The past versions of R-CNN, from selective search (Girshick et al., 2014) to RPN, were all intended to increase training and reduce energy consumption. Although the development of RPN enables sharing of convolution values, YOLO uses an end-to-end method for object detection using an entire image as the input of the neural network to predict the coordinate position of the bounding box directly. YOLOv1 is fast in calculation and can be applied to real-time fields, but the prediction of the position is not accurate enough, and the performance of small object fields is poor. In addition, for object images' recognition, it is impossible to distinguish between the foreground and background of the object effectively. Interestingly, YOLOv2 (Redmon and Farhadi, 2016) imported the anchor box to increase accuracy. The original YOLOv1 version divides the image into 7 × 7 grids, and each grid predicts two bounding boxes, which is better than importing 1,000 pre-selected regions into the anchor box. The fully connective layer was removed and changed to a fully convolutional network, and dropout was removed to optimize the overall speed and accuracy of YOLOv3 (Redmon and Farhadi, 2018). The maximum input of the image can reach 608 × 608 pixels, and many optimizations have been made. For example, residual neural network (ResNet) and feature pyramid network (FPN) are used to improve the detection of small objects; the darknet53 network is applied; the detection threshold of YOLO model can be adjusted in the training process according to the threshold parameter in its network architecture. Faster R-CNN's architecture RetinaNet is built using ResNet. Comparing YOLOv3 with ResNet, it can be observed that YOLOv3 can achieve the same results in a relatively short time. The mentioned FPN architecture uses three boxes of different sizes. The model can learn the image characteristics of different blocks through these three scales to improve YOLO's shortcomings in small object prediction (Redmon and Farhadi, 2018). YOLOv4 (Bochkovskiy et al., 2020) has improved the previous version in many aspects. The author uses the Mosaic method, which used random scaling and cropping to mix and stitch 4 kinds of pictures from the original datasets, to enrich the data set and enhance the stability of the model for small target detection. For stability, the network uses CSPDarknet53, which is composed of darknet53 and CSPNet (Wang et al., 2019), which greatly reduces equipment requirements and computing speed. The author also drew on the PANet (Path Aggregation Network) (Liu et al., 2018) used in the field of image segmentation, integrates PAN on the basis of the FPN architecture, and adds SPP (Spatial pyramid pooling) to improve the ability of feature extraction.

## Materials and Methods

**Figure 1:**
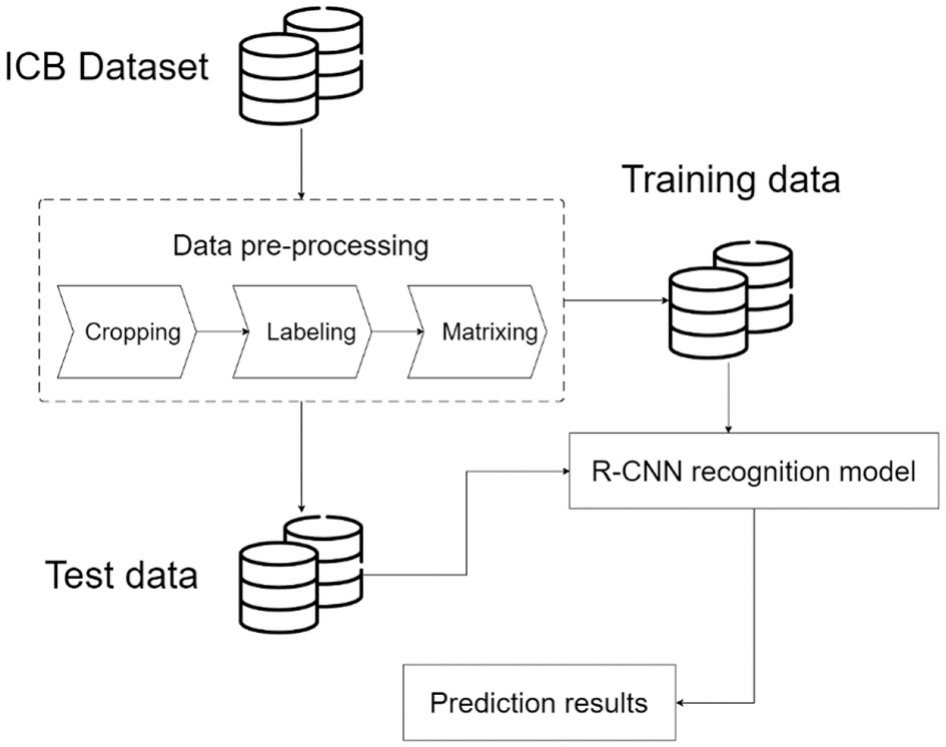
Image recognition and object detection model for ICB.

Nowadays, in implementing smart manufacturing, intelligence should be implemented to achieve the most effective results to complete the quality management part of FMS effectively. In the field of traditional non-intelligent manufacturing, several problems are encountered. (1) Although the current automatic optical inspection method can achieve accurate inspection, its parameter setting is too strict, resulting in a pass rate of ~70%. It is still necessary to employ field operators to complete the second inspection stage to ensure the yield. (2) In traditional manual monitoring, the biggest problem is that people may suffer from mistakes due to inattention or fatigue, which affects the quality of some parts. (3) In the field of smart manufacturing, the inspection process should give high accuracy in real-time. Therefore, we must find a suitable image recognition model to apply here. The deep learning image recognition method allows the selected model to learn the item's features by using the features provided in the dataset. Consequently, accurate image recognition in the manufacturing system can be attained, and the integrity and quality inspection of ICBs can be completed through precise image recognition. This study aims to build an image recognition model of ICBs so that various types of ICBs can be classified in this model according to the system architecture flow of this study, such as Figure 1. In the image recognition and object detection model for ICB, the first stage is to collect part of the dataset and establish the image database standard that can be used based on the R-CNN method. Then, the collected images are cropped, feature labeled, and matrixed. Later, the dataset is divided into training data and test data. Next, an R-CNN is constructed to train the image recognition model. Finally, the image of the test data is mapped to the recognition model to generate the result. The results are respectively sent to the user and the server end for data analysis applications.

### ICB Data Collection

The training data in this study has five types of ICB images, and 100 images are collected based on these five types. The ICB images used in this study must contain identifiable features under specified conditions. First, training the model for collecting images is standardized to better sample the image features in the data collection part. While collecting images, two methods of data collection can be used. In both scenarios, the ICB that needs to be pictured must be placed in the center of the image and then divided into near and far for feature collection. Moreover, in the collection process, the background is changed to be used for image recognition under different backgrounds. The focus of long-range shooting has covered the entire ICB. On the contrary, the focus of short-range shooting is mainly on the integrity and clarity of the internal structure of the ICB. Both methods must sample the different angle characteristics of the ICB during the shooting process. At least 100 samples of each category must be tested, and the final data collection shall be based on the five types of ICBs.

### Pre-process

To successfully import the dataset into the model's training process, pre-processing must be done. The purpose of data pre-processing is to keep the input data in a consistent form, such as fixed image size or labeling so that it fits within the processing range of the R-CNN model before entering the model training process. The pre-processing of the data here includes three steps: the first step is to cut each ICB dataset into the size of 1,024 × 1,024 pixels without losing key details of the board. Only then can the dataset be easily imported into the model. The second step is to mark the image area through the open-source software Labellmg. Labellmg is the most commonly used software for labeling images. For our classification, we can mark the features in the image by selecting the box. The third step is to carry out matrix work. The image recognition model is different from humans. Humans capture features through images viewed by their eyes. Machines, on the other hand, use a data matrix to understand the key features in blocks in two-dimensional images and then use this matrix in the model for the application.

To train the YOLO model more effectively, pre-processing must be carried out for the first stage of data collection. The purpose is to make the model more focused on learning features with organization and clarity when learning images. In this stage, we must first set a fixed image size to mark the learning features of the model and then, convert the marked features into a matrix to train the neural network model. The steps are as follows:

- Image cutting: Use ImageSplitter, an online image cutting tool on the Internet, to fix the image size to capture the characteristics of each image and define the fixed size as 1,024 × 1,024 pixels.
- Data label matrix: Use the open-source software Labellmg to label images and feature matrix for training the model to correspond to the features that this study hopes to learn to complete the full model training.

### Model Selection

**Figure 2:**
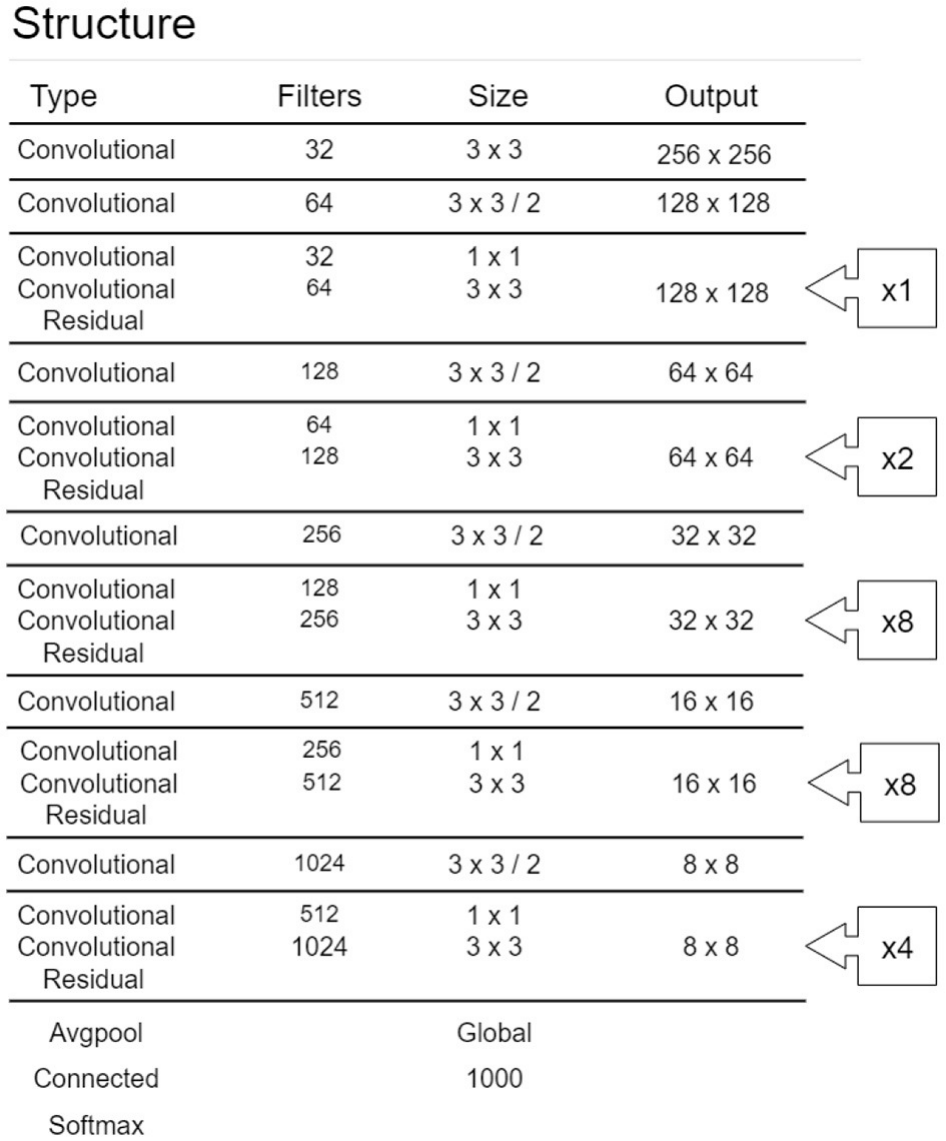
YOLOv3_Darknet53 network (Redmon and Farhadi, 2018).

There have been many studies comparing model suitability for smart manufacturing. In this study, YOLO is selected as the model. In the past, when recognizing R-CNN in images, most of them used the model architecture of Faster-RCNN for image recognition. Indeed, the accuracy of Faster-RCNN is still the highest, but to deal with the field of smart manufacturing, real-time recognition of images is vital. YOLO has a faster real-time response speed with an accuracy of results close to Faster-RCNN. Therefore, this study uses YOLO as the R-CNN model of the architecture. Figure 2 shows YOLO's network configuration diagram (Redmon and Farhadi, 2018). YOLO is a multi-level R-CNN, where the first layer defines the dimensions of the input parameters and the output layer performs classification actions according to its final output results. Thus, the hidden layer between the input and output layers is the main structure of this R-CNN. The activation function used after each CNN layer is Leaky ReLU, and Residual refers to the ResNet architecture, which replaces the activation function covering the two-layer CNN. The functions and tasks of each layer are as follows:

- Input layer: After an ICB image is cut into the input size of the model, the learning features are marked. Then the parts are converted into a matrix pattern that the machine can understand, thereby becoming the model's input data.
- Convolution layer: The ICB image is two-dimensional in this study, so a two-dimensional convolutional layer is used. The convolutional layer can parameterize the image of the ICB through the size of its image, the kernel size, and the feature factor.
- Leaky ReLU layer: This derivation of ReLU uses the function in the neural network node to increase the non-linear characteristics of the entire neural network function and define the node's output so that it is suitable for solving the dying ReLU problem.
- Residual layer: Its original name is the residual network (ResNet) and its core is residual block. To solve the problem of an unexpected increase in the error rate during training, some of the weight parameters may tend to zero or become zero during the regular conversion of each layer, and the error rate will increase when the best solution is ignored.
- Average pooling layer: This layer replaces the fully connective layer used at the end of the general neural network. The most significant disadvantage of the fully connective layer is that the number of parameters is too large, resulting in overfitting. Therefore, the average pooling layer replaces the weighted connection layer to directly give each feature its sense to prevent the overfitting problem caused by the fully connected layer.
- Softmax layer: The Softmax layer multiplies the weight matrix, adds the characteristic error to generate the Softmax function, and applies it to the output of the average pooling layer.
- Classification layer: The classification layer obtains the output of the previous Softmax layer and classifies the input data according to the final output.

This study is built on four models based on YOLOv3, namely, YOLOv3, YOLOv3_tiny, YOLOv3_voc, and YOLOv3_spp. The comparison of these four models is shown in Table 1.

- YOLOv3: It is the third version of the initial model of YOLO, which adds the model architecture of Darknet53 and a multi-scale method to verify the feature map. The multi-scale approach helps the model learn the detailed features of the image through three different sizes, which is a breakthrough for YOLOv3. In addition, it can use images up to 608 × 608 as input data (Redmon and Farhadi, 2018).
- YOLOv3-tiny: There are 19 layers of CNN, which is a part of the gap compared with the 75 layers of the original version. Its advantage is that it has better applications for devices with limited computing resources and fast training.
- YOLOv3-voc: It is an improvement of YOLOv3. The original input of YOLOv3 is 608 × 608, and YOLOv3-voc is 416 × 416, which is the same as that of YOLOv3-tiny. This method focuses on retaining the convolutional layer, reducing the image size to improve the training speed, and reducing its ignore thresh (the threshold value that the overlapped block of the predicted labeled area and the overlapped labeled area must exceed) for training.
- YOLOv3-spp: The purpose of adding the SPP to YOLOv 3 is to convert the selected feature maps to the same size using the SPP fixed-scale conversion method to achieve a more accurate learning feature model training (Huang and Wang, 2019).

**Table 1:** Comparison table based on YOLO model.

**Model**	**Advantages**	**Disadvantages**
YOLOv3	Benchmark	Benchmark
YOLOv3_tiny	Fast training and lightweight architecture	The number of model layers is low, and it is difficult to reach the maximum value
YOLOv3_voc	Low confidence threshold and small input image	Features are relatively easy to lose focus
YOLOv3_spp	Can be used with the multi-scale conversion of eigenvalues	Features are easily compressed during conversion

### Model Adjustment

**Figure 3:**
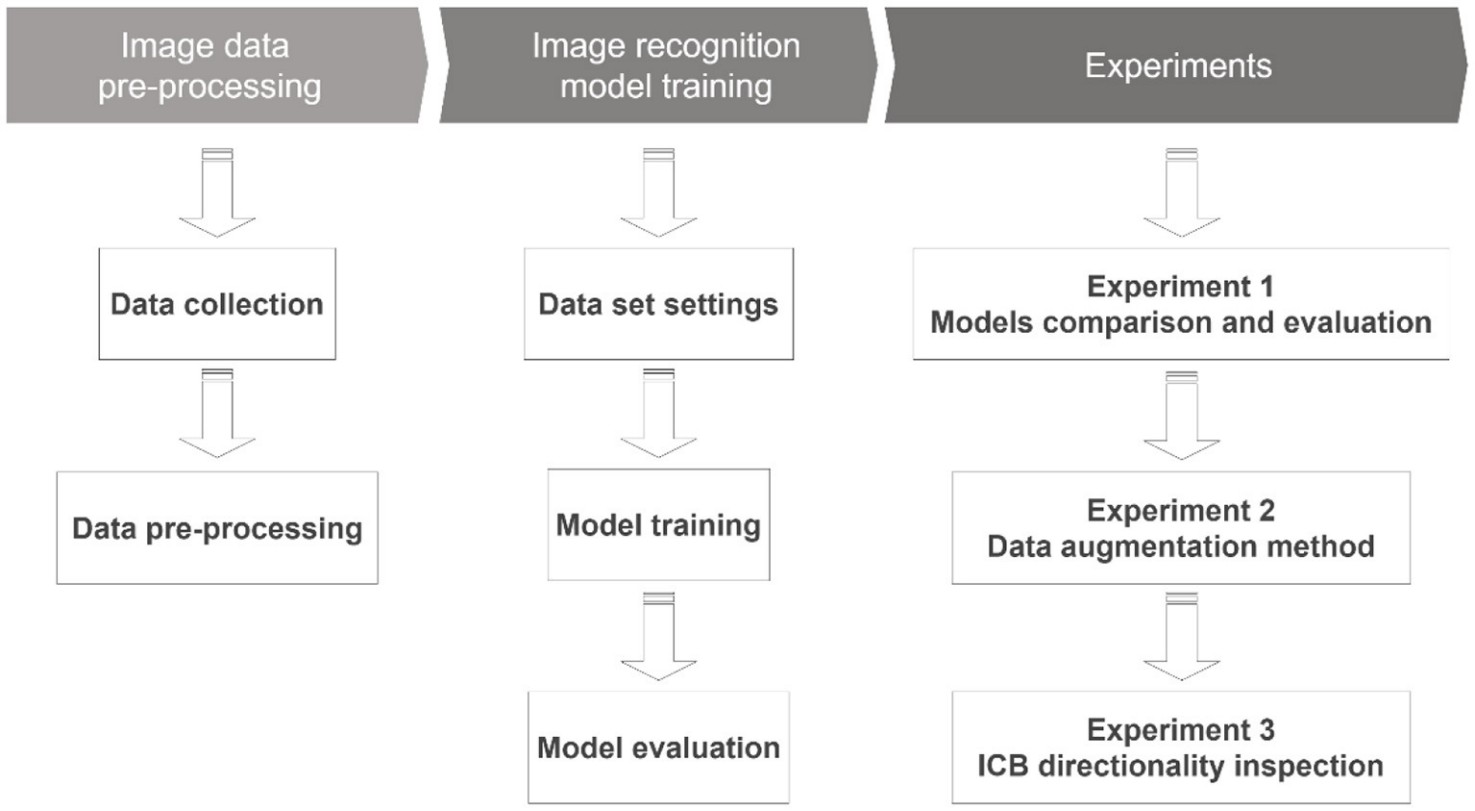
Research flow chart.

YOLO's overall training process includes classification design, training dataset cutting, test dataset cutting, naming of each category, and parameter settings in order. These five items are YOLO's current framework, and the selection and setting of the datasets and models are used to complete the image recognition work. In this process, the related settings of model adjustment are introduced as follows: Classes: identify target types; Train: training dataset settings; Valid: verify dataset settings; Names: specify the name of the target type; and Backup: store model parameters. During the model training process, YOLO trains the recognition model based on the training data. After repeated iterative training, the image recognition and object detection results are generated according to the model parameters and the classification settings. This result has the characteristics of the relevant image data in the learning process. Finally, the membership classification is marked when an output is achieved, and the overall recognition accuracy is returned. The parameter setting values when using the learning model in this study are as follows. (1) Batch: 16 (refers to the number of batches that have passed to update the parameters once); (2) Subdivisions: 4 (if the memory is insufficient, the batch will be divided into sub-batches); (3) Width: 608 (the width of the input image data); (4) Height: 608 (the height of the input image data); and (5) Momentum: 0.9 (in neural networks, it is a variant of the stochastic gradient descent. It replaces the gradient with a momentum, which is an aggregate of gradients); (6) Decay: 0.0005 (parameter weight attenuation setting to prevent overfitting); and (7) Learning rate: 0.001 (initial learning rate). The study process includes data collection, pre-processing methods, experimental environment, model establishment, discussion, evaluation, and analysis to verify the proposed R-CNN image recognition model design method applied to the smart manufacturing field. Figure 3 shows the flow chart of the study.

## Experiments

### Evaluation Metrics

(1) Mean Average Precision (mAP): As shown in equation (2), the accuracy of all classifications is averaged (an average is calculated by estimating the prediction and actual accuracy). The basic accuracy calculated is as follows:

TP(ICB1), True Positive in ICB1: The classification result of the current model is correct, and the overlap between the predicted labeled area and the actual labeled area is high enough.

FP(ICB1), False Positive in ICB1: The classification result of the current model is incorrect, or the overlap between the predicted labeled area and the actual labeled area is not high enough.

From this, the accuracy of classification ICB1 can be calculated from the following equation (1):


(1) $$Precision(ICB1)\;=\;\frac{TP(ICB1)}{TP(ICB1)+FP(ICB1)}$$

Therefore, the mAP of each category is calculated from equation (2) (take *N* categories as an example):


(2) $$\begin{array}{r} mAP\;=\;\frac{Precision\left(ICB1\right)+\ldots +Precision\left(ICBN\right)}{N} \end{array}$$

(2) Recall: The ratio of the number of correctly identified categories in the prediction result to the target in the test data, calculated from equation (3).


(3) $$Recall\left(ICB1\right)\;=\;\frac{TP(ICB1)}{TP\left(ICB1\right)+FN(ICB1)}$$

TP(ICB1), True Positive in ICB1: The classification result of the current model is correct, and the overlap between the predicted labeled area and the actual labeled area is high enough.

FN(ICB1), False Negative in ICB1: It means that the current model test set is not classified in the pre-set classification, and the recognition model classifies it as one of the classifications.

### Experimental Designs

This study uses the evaluation indicators of the YOLO image recognition model to compare the image recognition results of four different models of YOLO and enhance the difference in the size of the training data through the image augmentation fusion method. The following three aspects are used to evaluate the performance of the proposed method.

- Models comparison and evaluation: This study identifies four different models based on YOLOv3 and use fixed parameters to train the model. In addition, five different types of ICB images are used; each type has 100 images, with 80 of them used for training and 20 for verification. Thus, the total dataset contains 400 training images and 100 verification images. Finally, an additional 60 images are used as a test.
- Data augmentation: In this stage, each classification's original ICB images are used for data augmentation methods. The amplification parameters used are rotation_range, width_shift_range, height_shift_range, shear_range, zoom_range, horizontal_flip, vertical_flip, and fill_mode. The 100 original images of each classification are processed by the data augmentation method to generate 500 images, and then 400 images per classification are used as the model's training data. The remaining 100 images are used as verification data. There are a total of 1,600 training images and 400 verification images. Finally, the same 60 test data are used to discuss the analysis of the data augmentation method for the model feature training and learning.
- ICB directionality inspection: This stage of the experiment checks the core image of the integrated circuit board to see whether the chip is installed incorrectly. Type 5 of the ICBs is used to perform this test. The whole experiment uses 88 training images 22 verification images, and 50 test images. These images contain both correct and incorrect integrated circuit images (incorrect images are ICBs with wrong core directionality). The images are inspected to see whether the model can correctly check the core installation error of the ICB. This experimental model uses the best model discussed in the 1st and 2nd experiments for training.

### Training Dataset

**Figure 4:**
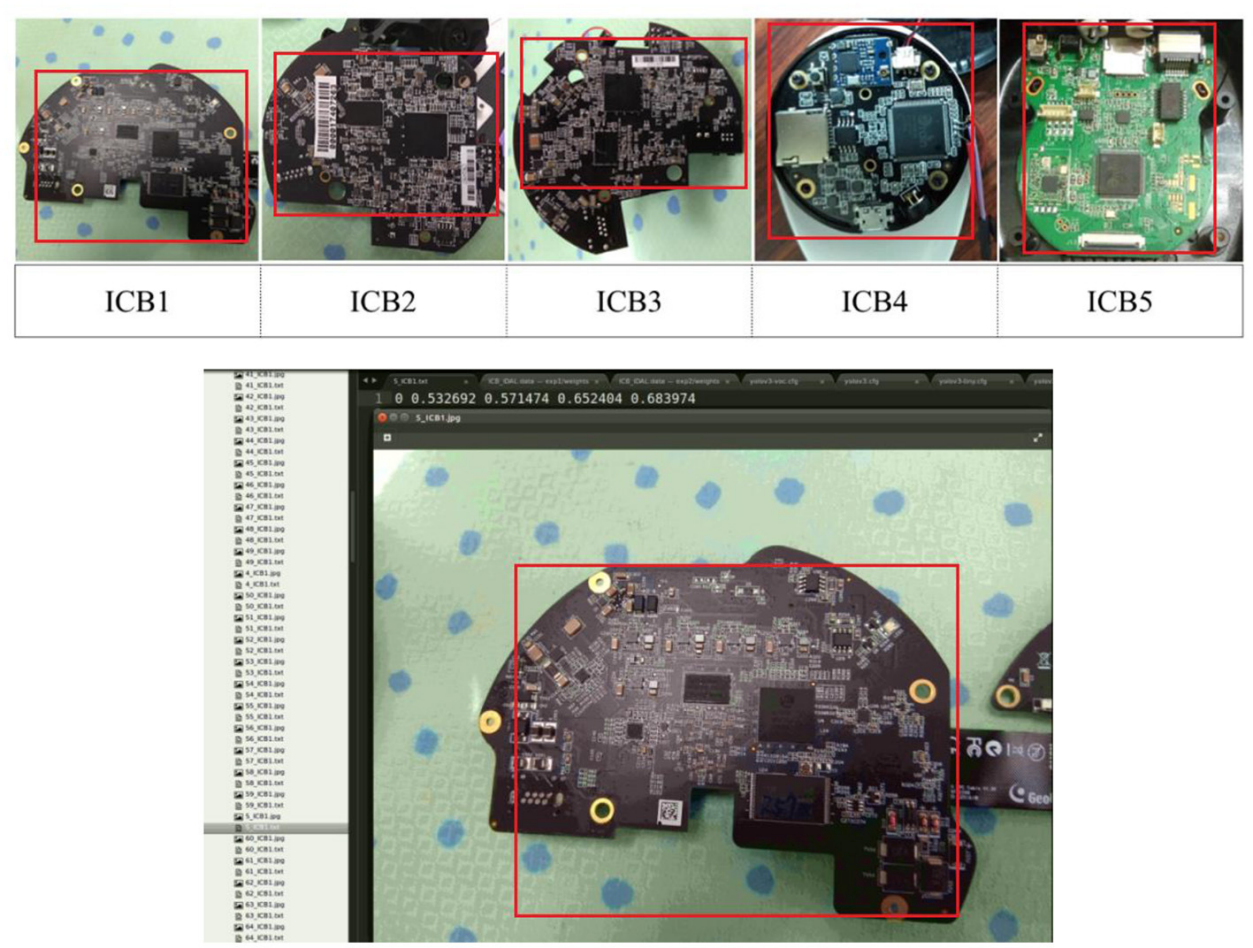
ICB type table and image labeling.

As shown in Figure 4, this study collects 100 images of each of the five types of ICB, and the data must be labeled during YOLO training. After labeling each image, the image is set to the learning format of the YOLO model on the Darknet platform corresponding to its classification. Images of each format are divided into training and verification data to complete the preliminary model training settings.

### Experimental Results

#### Models Comparison and Evaluation

During the model training process, we use the YOLOv3 model with the parameters that have been set, and the training iteration target is 10,000. During the training, the values are stored as train_log_loss.txt file to help us understand each iteration's error value and average error, the current learning rate, the number of training images, and the training time. The entire training set includes 500 ICB images, which are classified into five categories, of which 100 ICB images are used as the training phase verification of the overall model, and the number of training iterations is 10,000. Then, using the trained model parameters, the current classification status of each classification and the generation of mAP and recall of the model are calculated through the additional 60 images of test data. In this stage, the four models YOLOv3, YOLOv3-tiny, YOLOv3-voc, and YOLOv3-spp are presented in sequence from Case 1 to Case 4, respectively, showing the training process and the accuracy during training and the final test accuracy. Experimental discussion in Table 2 shows that the YOLOv3-voc model is significantly better than the other three in 10,000 iterations. Experiment 1 shows that the YOLOv3-voc model is the best, and its overall average error is 0.036, and its maximum average accuracy is 91.9%.

**Table 2:** Experiment 1-various YOLO models result comparison table.

**Model**	**Average iteration time**	**Average error**	**Average training accuracy**	**Training recall rate**	**Average test accuracy**	**Test recall rate**	**Maximum average accuracy**
YOLOv3	0.94	0.05	98.87%	98%	91.39%	88%	91.39%
YOLOv3-tiny	0.18	0.203	98.82%	99%	91.63%	87%	91.73%
YOLOv3-voc	0.72	0.036	99%	100%	91.66%	90%	91.9%
YOLOv3-spp	0.98	0.057	98.47%	99%	87.86%	87%	88.68%

##### Case 1: YOLOv3

**Figure 5:**
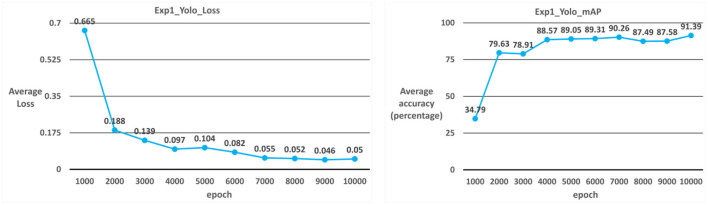
YOLOv3 average error rate and average accuracy rate.

The model used in Case 1 is the YOLOv3 model. As shown in Table 2; Figure 5, the average iteration time is 0.94 s, and the average error rate is 0.05. Therefore, the average accuracy rate in the training phase can reach 98.87%, and the recall rate can reach 98%. During the test phase, 60 ICB images are used as test data. As a result, the average accuracy rate in the test phase can reach 91.39%, and the recall rate can reach 88% due to the overall model performance.

##### Case 2: YOLOv3-tiny

**Figure 6:**
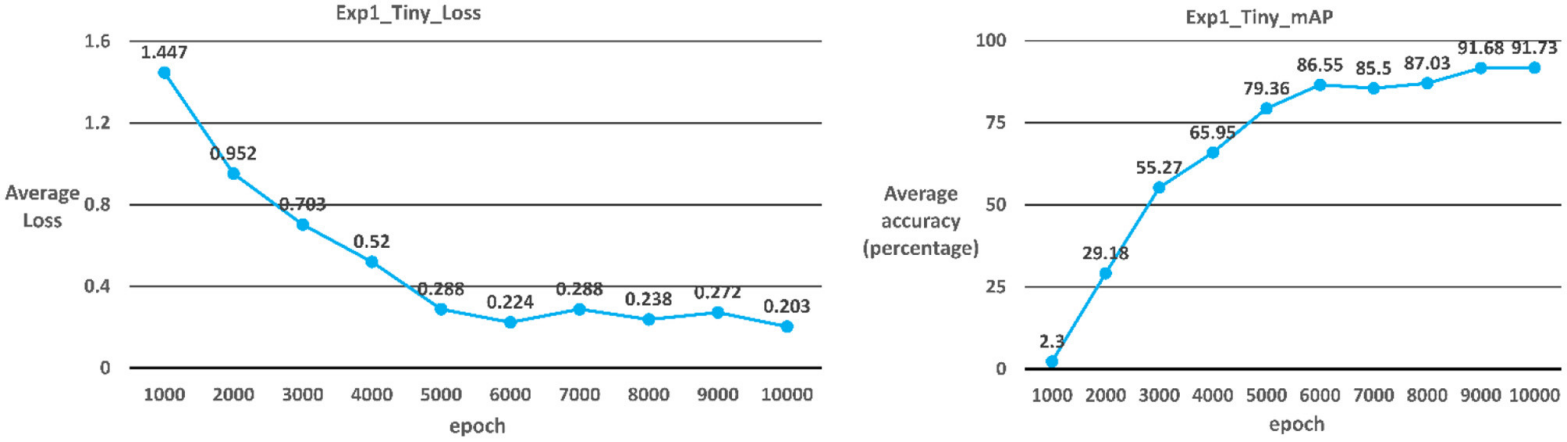
YOLOv3_tiny average error rate and average accuracy rate.

The model used in Case 2 is the YOLOv3-tiny model. As shown in Table 2; Figure 6, the average iteration time is 0.18 s, and the average error rate is 0.203. Therefore, the average accuracy rate in the training phase can reach 98.82%, and the recall rate can reach 99%. During the test phase, 60 ICB images are used as test data. As a result, the average accuracy rate in the test phase can reach 91.63%, and the recall rate can reach 87% due to the overall model performance.

##### Case 3: YOLOv3-voc

**Figure 7:**
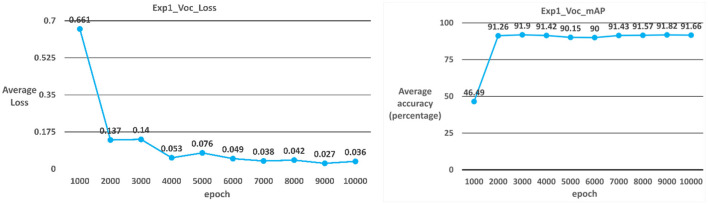
YOLOv3_voc average error rate and average accuracy rate.

The model used in Case 3 is the YOLOv3-voc model. As shown in Table 2; Figure 7, the average iteration time is 0.72 s, and the average error rate is 0.036. Therefore, the average accuracy rate in the training phase can reach 99%, and the recall rate can reach 100%. During the test phase, 60 ICB images are used as test data. As a result, the average accuracy rate in the test phase can reach 91.66%, and the recall rate can reach 90% due to the overall model performance.

##### Case 4: YOLOv3-spp

**Figure 8:**
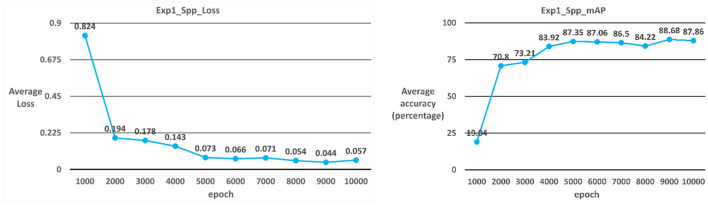
YOLOv3_spp average error rate and average accuracy rate.

The model used in Case 4 is the YOLOv3-spp model. As shown in Table 2; Figure 8, the average iteration time is 0.98 s, and the average error rate is 0.057. Therefore, the average accuracy rate in the training phase can reach 98.47%, and the recall rate can reach 99%. During the test phase, 60 ICB images are used as test data. As a result, the average accuracy rate in the test phase can reach 87.86%, and the recall rate can reach 87% due to the overall model performance.

#### Data Augmentation

**Figure 9:**
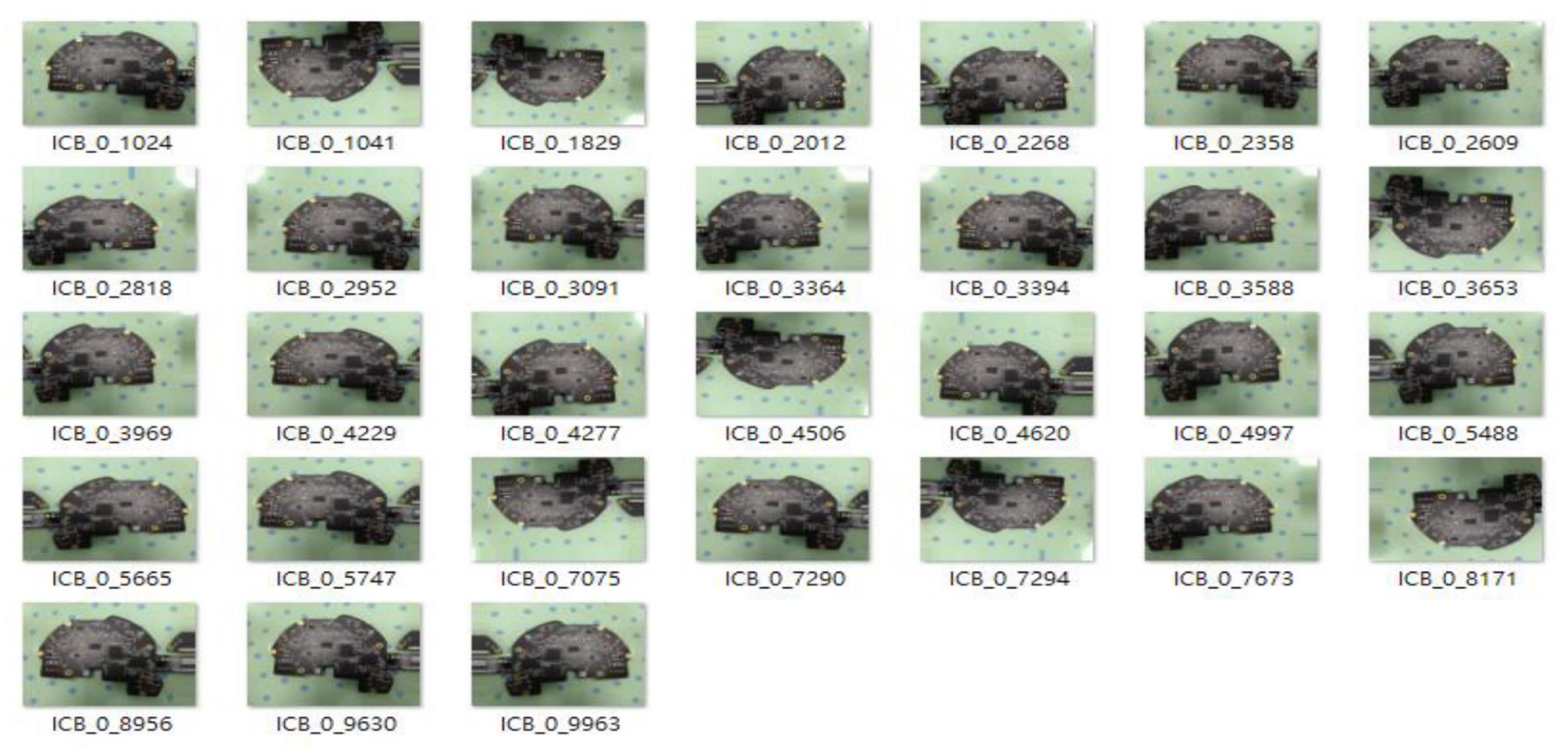
Results of data augmentation methods.

In this stage of the experiment, the impact of the amount of data on training is discussed in advance, so data augmentation methods are used to increase the dataset. The result of a single image using the data augmentation method is shown in Figure 9, and the image generated by the data augmentation method still requires data pre-processing and labeling.

In this stage of the experiment, as shown in Table 3, the performance of the YOLOv3-voc model at the number of iterations of 10,000 is significantly better than the other three. The experimental result of experiment 2 is that the YOLOv3-voc model is the best. It has an average error value of 0.06, and the highest average accuracy rate can reach 96.53%.

**Table 3:** Experiment 2-various YOLO models result comparison table (after data augmentation).

**Model**	**Average iteration time**	**Average error**	**Average training accuracy**	**Training recall rate**	**Average test accuracy**	**Test recall rate**	**Maximum average accuracy**
YOLOv3	0.67	0.105	99.62%	99%	91.14%	92%	94.86%
YOLOv3-tiny	0.20	0.251	99.55%	97%	93.56%	90%	94.87%
YOLOv3-voc	0.71	0.06	99.8%	100%	94.72%	95%	96.53%
YOLOv3-spp	0.66	0.079	97.07%	97%	92.22%	95%	94.58%

Comparing the results from the YOLOv3-voc model of experiment 1 and experiment 2, listed in Tables 2, 3, respectively, it is found that using data augmentation methods to allow the model to learn more image features can significantly improve its average accuracy and recall rate.

#### ICB Directionality Inspection

**Figure 10:**
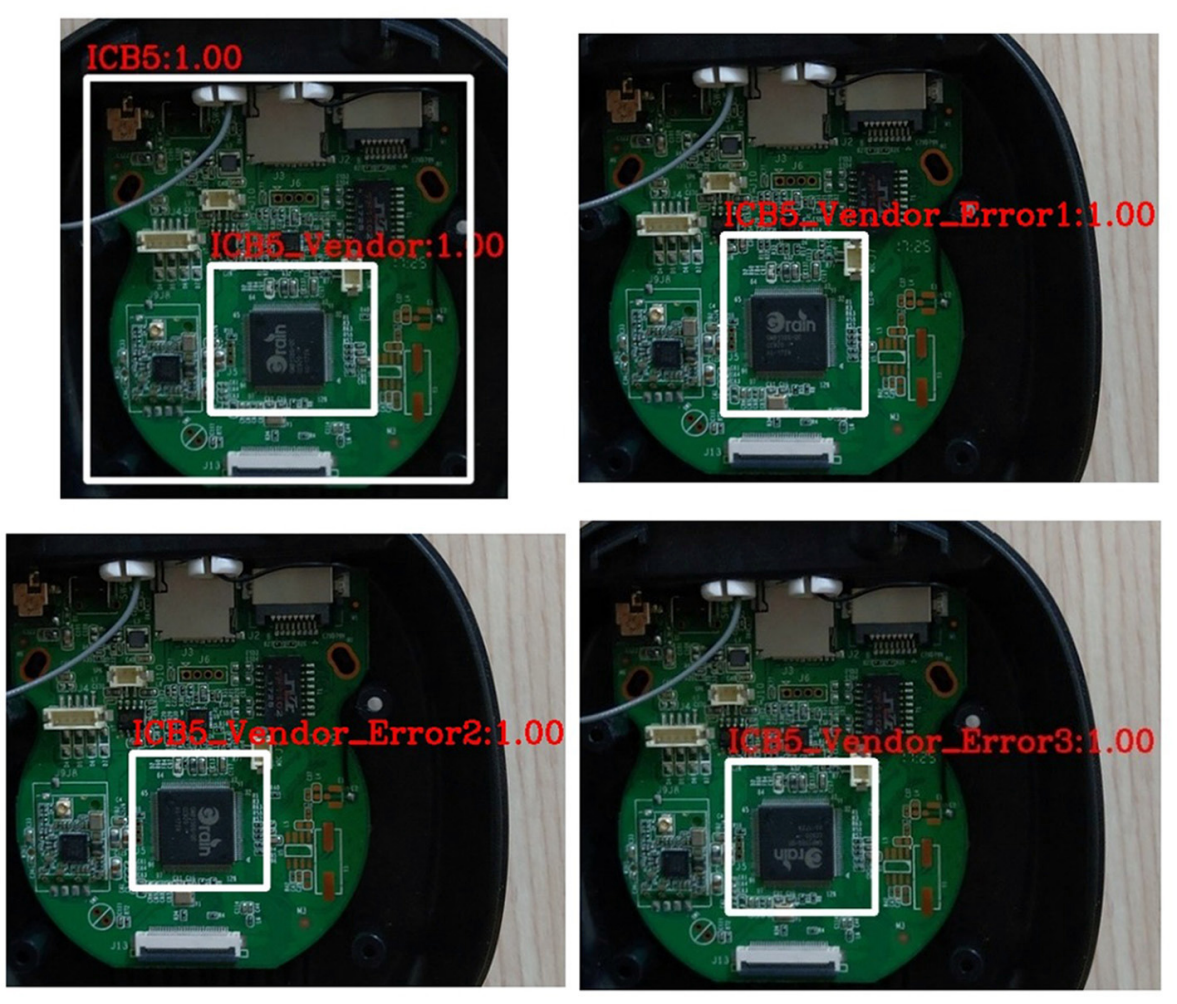
Directionality classification and inspection of integrated circuit image (correct, error type 1, error type 2, and error type 3).

A total of 160 images of type-5 integrated circuit board model (ICB5) are used in this experiment stage. In the experiment, the images are divided into 88 for training datasets, 22 for verification datasets, and 50 for test datasets; all datasets contain both correct and incorrect integrated circuit images. The model used is the YOLOv3_voc model, and the model is trained to 10,000 iterations. The identification results are shown in Figure 10, showing the correct identification and three kinds of incorrect identification. Correct: The direction of the ICB recognition image is correct; Error type 1: The direction of the ICB recognition image shows type one error; Error type 2: The direction of the ICB recognition image shows type two error; Error type 3: The ICB recognition image direction shows type three error; None: Cannot identify the direction of the ICB identification image. For the result, among the 50 test images, only one image is currently not recognized. The original training model and actual prediction results are shown in Table 4, showing a correct rate of 98%, which is more than 90% required for general applications. Furthermore, the recognition time for each image is no more than one s, which is practical for smart manufacturing fields that require real-time recognition.

**Table 4:** Confusion matrix of ICB Image directionality recognition results.

**Original predict**	**Correct**	**Error 1**	**Error 2**	**Error 3**	**None**
Correct	30	0	0	0	0
Error 1	0	6	0	0	0
Error 2	0	0	7	0	0
Error 3	0	0	0	6	0
None	0	0	0	1	0

## Summary

The experiment in this study is divided into four stages. In the first stage, we must execute the pre-processing of the dataset to complete the learning goal and then generate a complete training process. The second stage focuses on the four models under YOLOv3 to explore more suitable model for smart manufacturing. In the third stage, the influence of the image augmentation fusion method on the identification results of the model is discussed based on the comparison results of the second stage. Finally, the fourth stage discusses the application of its model in the actual field. The results of the experiment show the following conclusions:

### YOLOv3 Model Selection

In the experimental part of this study, because we hoped to apply the model to smart manufacturing and because the advantage of YOLO is the speed of image recognition, so we hoped to choose a model with excellent training cost and actual recognition results. After comparing YOLOv3, YOLOv3-tiny, YOLOv3-voc, YOLOv3-spp under the third version of YOLO, the experimental results show that YOLOv3-voc is the best choice, which can reach the highest 96.53% accuracy rate and 94.72% average accuracy rate during test stage under the experimental conditions, the performance is quite good. Although the second-place YOLOv3-tiny model also has an average accuracy of 93.56, the difference in training time to reach the same level is quite large, so the final selection of the model is YOLOv3-voc. Of course, if we further optimize various parameters or lengthen the overall training time, it is possible to obtain higher accuracy.

### Effectiveness of Data Augmentation Methods

In the second model comparison, this study applied a data augmentation method to the dataset to increase the data size and learn more features. Among them, data augmentation methods include angle flipping, focus scaling, and image cropping. As a result, the size of the dataset increased from 100 images to 400 images. Thus, the original average accuracy rose from 91.66 to 94.72%, which proved that the model has a higher grasp of the image characteristics of the ICB after using the image augmentation fusion method.

### Application of Directional Inspection of the Integrated Circuit Board

This study focuses on the actual image recognition of the ICB. We used the brand image of the ICB as the inspection target to determine the correctness of its installation direction. After experimental testing, a total of 160 images were used to complete the training test. In the last 50 test images, the detection accuracy rate reached 98%, exceeding the 90% threshold in general actual application environment, proving that the model could be used for application testing.

### Discussion on the Number of Iterations of the YOLOv3_Tiny Model

This study also had a separate discussion on the YOLOv3_tiny model. The training cost of the model and the experimental data of the YOLOv3_tiny model are discussed in the first few subsections. Compared with other models, the training time is shorter due to its lightweight architecture. Although a high level of average test accuracy can be achieved through multiple training iterations, the overall time cost is still slightly higher than YOLOv3_voc. Nevertheless, its advantage is that the equipment is relatively standard, and it is easy to train a good model for application quickly.

## Conclusions

Smart manufacturing must cover functions such as automated information perception, automated decision-making, and automated execution. What drives these automated processes rely on data and every piece of this data comes from various sensors, and image recognition is one of the methods that can be used. Moreover, based on the deep learning architecture, the work can be completed by the trained model. The results prove that YOLO's model can achieve the lowest model training cost in an automated environment that requires image recognition speed and excellent image recognition results using the ICB image under the pre-processing method of this study. Thus, the model is quite suitable for application in the smart manufacturing field, effectively achieving automatic perception.

This study also discusses several YOLO models. Among them, YOLOv3_voc has the best performance, with the highest accuracy rate of 91.9%. When combined with the pre-processing in experiment 2 of this study using the image data augmentation fusion method, the highest accuracy can reach 96.53, 4.5% higher than the original model without the data augmentation method. In the final experiment, the image of the ICB was used and the directional inspection accuracy could reach 98%, which met the 90% threshold required in general application. In addition, given the real-time nature of the production site, this study takes <1 s to identify each image, which can be a good candidate for application with real-time requirements. This proves the feasibility and accuracy of R-CNN in the field of smart manufacturing.

Regarding the research limitations in this study, since it is impossible to collect all different ICB image data, the ICB image data sources in this study are only specific to five different types of webcams. In addition, in terms of model selection, the YOLOv3 model was used in this study in consideration of both machine performance and accuracy. In the future, more innovative models and more various ICB image data can be used in this architecture. In addition, to optimize the parameters of this model for the future development of this study, the biggest problem is actually the availability of data. Although the R-CNN can achieve excellent image recognition results, it requires many data behind it and must be labeled as learning features. To achieve the ultimate automatic perception, automatic correction is needed. The automatic correction introduced in image recognition provides new data that can be imported into the dataset of the model for learning. If it could be improved, the results of the learning are believed to be more prominent. Another part is about the method of image pre-processing. Although this study uses image data augmentation fusion methods, it may be possible to import binary image processing to increase data in the future.

Finally, we hope the model can be applied to smart manufacturing as practical application to make overall learning adjustments. There will be some problems in the actual field, such as the effect of light that may cause reflections when the ICB image is automatically detected, resulting in unrecognizable results. Therefore, it may be necessary to sample the characteristics of the ICB itself and some other features to assist the image recognition process.

## Data Availability Statement

The raw data supporting the conclusions of this article will be made available by the authors, without undue reservation.

## Author Contributions

S-YL contributed to conception, design, formal analysis, formulated methodology, funding acquisition of the study, and reviewed and edited the manuscript. S-YL and H-YL organized the data curation and wrote the first draft of the manuscript. H-YL analyzed the image data. All authors contributed to manuscript revision, read, and approved the submitted version.

## Funding

This work was supported by the Ministry of Science and Technology of Taiwan under Grant MOST 109-2410-H-197-002-MY3.

## Conflict of Interest

The authors declare that the research was conducted in the absence of any commercial or financial relationships that could be construed as a potential conflict of interest.

## Publisher's Note

All claims expressed in this article are solely those of the authors and do not necessarily represent those of their affiliated organizations, or those of the publisher, the editors and the reviewers. Any product that may be evaluated in this article, or claim that may be made by its manufacturer, is not guaranteed or endorsed by the publisher.
